# Functional limitations of people with rheumatoid arthritis or axial spondyloarthritis and severe functional disability: a cross-sectional descriptive study

**DOI:** 10.1007/s00296-023-05487-z

**Published:** 2023-11-25

**Authors:** Max M. H. Teuwen, Salima F. E. van Weely, Thea P. M. Vliet Vlieland, Thom Douw, Dirkjan van Schaardenburg, Alfons A. den Broeder, Astrid M. van Tubergen, Maria A. T. van Wissen, Cornelia H. M. van den Ende, Maaike G. J. Gademan

**Affiliations:** 1 Department of Orthopaedics, Rehabilitation and Physical Therapy, Leiden University Medical Center (LUMC), Albinusdreef 2, P.O.Box 9600, 2300 RC Leiden, The Netherlands; 2 University of Applied Sciences Leiden, Leiden, The Netherlands; 3 Department of Rheumatology, Center for Rehabilitation and Rheumatology, Reade, Amsterdam, The Netherlands; 4 Department of Rheumatology, Sint Maartenskliniek, Nijmegen, The Netherlands; 5 Department of Rheumatology, Maastricht University Medical Center, Maastricht, The Netherlands; 6 Care and Public Health Research Institute (CAPHRI), Maastricht University, Maastricht, The Netherlands; 7 Department of Rheumatology, Radboud University Medical Center, Nijmegen, The Netherlands; 8 Department of Research, Sint Maartenskliniek, Nijmegen, The Netherlands; 9 Department of Clinical Epidemiology, Leiden University Medical Center (LUMC), Leiden, The Netherlands

**Keywords:** Rheumatoid arthritis, Axial spondyloarthritis, International classification of functioning, Disability and health, Patient-reported outcome measures, Difficult-to-treat

## Abstract

The objective of the study is to describe the nature of functional limitations in activities and participation in people with Rheumatoid Arthritis (RA) or axial SpondyloArthritis (axSpA) with severe functional disability. Baseline data from people with RA (*n* = 206) or axSpA (*n* = 155) and severe functional disability participating in an exercise trial were used. Their three most limited activities were derived from the Patient Specific Complaint (PSC) instrument and linked to the International Classification of Functioning and Health (ICF). The frequencies of ICF categories were calculated and compared with Activities and Participation items of the ICF Core Sets for RA (32 second-level categories) and Ankylosing Spondylitis (AS) (24 second-level categories). In total 618 and 465 PSC activities were linked to 909 (72 unique in total; 25 unique second-level) and 759 (57 unique in total; 23 unique second-level) ICF categories in RA and axSpA. Taking into account all three prioritized activities, the five most frequent limited activities concerned the ICF chapter “Mobility”, and included “Walking” (RA and axSpA 2 categories), “Changing basic body position” (RA and axSpA 1 category), “Stair climbing”(RA) and “Grasping” (RA),“Lifting” (axSpA) and “Maintaining a standing position” (axSpA). In RA, 21/32 (66%) and in axSpA 14/24 (58%) unique second-level categories identified in the prioritized activities are present in the Comprehensive Core Sets. Most limitations of people with RA or axSpA and severe functional disability were seen in the ICF chapter “Mobility”. Most of the identified ICF categories were covered by the corresponding items of the ICF RA and AS Core Sets.

**Supplementary Information:**

The online version contains supplementary material available at 10.1007/s00296-023-05487-z.

## Introduction

Rheumatoid Arthritis (RA) and axial SpondyloArthritis (axSpA) are two prevalent forms of Inflammatory Arthritis (IA) and can both have a major impact on physical functioning, including limitations in daily activities and participation [1, 2]. The treatment consists of pharmacological and non-pharmacological interventions, with significant advancements in the pharmacological treatment options in recent decades [3, 4]. However, a subgroup of people with RA/axSpA has suboptimal treatment outcomes, which is reflected in the recent recognition of difficult-to-treat RA [5]. Some people with RA/axSpA still face severe functional disability despite optimal pharmacological treatment, stemming from joint damage accumulated over time, comorbidities or other health problems related to their rheumatic condition.

The optimal treatment of RA/axSpA requires shared decision-making between patients and clinicians, with goal-setting playing a crucial role [6, 7]. Literature on patient centered care emphasizes that treatment should address not only disease activity but also patients’ functional limitations [6, 7]. A cross-sectional study, involving people with RA, found that 62% of the patient–clinician pairs achieved concordance on prioritization of the treatment goal “have fewer problems doing daily activities” [8]. This highlights the importance of considering patients’ functional limitations when setting treatment goals. Despite the importance of addressing and prioritizing functional limitations as a treatment goal, there is limited literature on this topic. A systematic literature review, including 22 studies on treatment goal-setting for people with RA, identified functional limitations as a common theme within the physical experience of RA [9]. Goals on functional limitations included bending, engaging in physical activities and mobility [9]. However, none of the studies in that systematic review specifically included patients with severe functional disability. Such patients are likely to be represented in rehabilitation settings. In one study, a cross-cultural comparison between four countries of the contents of rehabilitation goals of people with RA admitted for rehabilitation was made [10]. In this, the rehabilitation goals were linked to the International Classification of Functioning, Disability and Health (ICF) [11] and ICF Core Set for RA [12], which includes the list of essential categories relevant to this specific health condition and health care context. It was found that most treatment goals were related to the ICF component “Activities and Participation” and fell within the chapters of “Mobility”, “Self-care”, and “Learning and applying knowledge” [10]. The contents of the rehabilitation goals were, to a considerable extend, covered by the Comprehensive ICF Core Set for RA [10]. However, the generalizability of the results to the current populations of people with RA/axSpA and severe functional disability may be limited [10]. This study was conducted ten years ago, in which (pharmacological) treatments have evolved and are more treat-to-target, the methods used to achieve treatment goals differed between countries and data are only available from people with RA.

Nowadays in the Netherlands, most people with RA/axSpA and severe functional disability requiring rehabilitative care are treated in primary care, with physical therapy being the most used intervention.

Currently, there are instruments available for goal-setting in treatment, such as an instrument developed for people with RA and clinicians [8]. Additionally, several goal-setting instruments suitable for rehabilitation settings have been evaluated in people with RA as well. These include the Rehabilitation Activities Profile (RAP) [13], the Canadian Occupational Performance Measure (COPM) [14], and the World Health Organization Disability Assessment Schedule 2.0 (WHODAS 2.0) [15].

Within the Dutch physical therapy community, the Patient Specific Complaint instrument (PSC) [16–20] is currently recommended. With the PSC, limitations in activities are identified and prioritized. The three highest ranked (and potentially modifiable) limitations in activities are scored on a 11-point numeric rating scale (anchors 0; no limitations—10; unable to perform) allowing evaluation over time [16–18].

Considering the limited knowledge regarding the nature of functional limitations of people with RA/axSpA and severe functional disability receiving physical therapy in primary care, this study aims to describe functional limitations in activities and participation of this subpopulation using the ICF as a reference. Insight into their prioritized functional limitations could facilitate the setting of treatment goals for daily activities.

## Methods

### Study design

This cross-sectional study concerns a descriptive analysis of the baseline data of two parallel randomized controlled trials (RCTs) investigating the effect of longstanding exercise therapy in primary care in people with RA or axSpA and severe functional disability (International Clinical Trials Registry Platform (ICTRP): Longstanding EXercise Therapy in patients with Rheumatoid Arthritis (L-EXTRA; NL8235) and Longstanding EXercise therapy in patient with axial SPondyolArthritis (L-EXSPA; NL8238)). All patients signed a written informed consent form and both studies were conducted in agreement with the Declaration of Helsinki (2013) [21]. The ethical approval was granted by the Medical Ethical Committee Leiden‐Den Haag‐Delft (METC LDD; L‐EXTRA: NL69866.058.19, L‐EXSPA: NL70093.058.19). Details of both studies were published previously [22]. For this analysis, baseline data from the included patients available on 14 February 2022 were used. The study was reported according to The Strengthening the Reporting of Observational Studies in Epidemiology (STROBE) Statement: guidelines for reporting cross-sectional studies.

### Participants

The inclusion and exclusion criteria of the RCTs have been published previously [22]. In brief, severe functional disability was defined as having self-perceived problems in performing basic activities of daily life (e.g. walking, dressing, washing oneself, using the toilet, preparing a meal, transfers). The problems should be related to their rheumatic condition, e.g. being due to persistent high disease activity, joint damage and/or deformities, complications of treatment, or co-morbidity. After patients had shown interest in the study, the presence of severe functional disability was to be confirmed during a structured telephone interview with one of the researchers (MT or MvW). In case of doubt, cases were presented and discussed in a larger team of researchers and clinicians to make the final decision on eligibility. If needed, additional information was requested from the patient or treating rheumatologist. After the screening, the treating rheumatologist was asked to confirm the diagnosis RA/axSpA of all eligible participants.

### Assessments

#### Sociodemographic and disease characteristics

The baseline sociodemographic and disease characteristics were collected using a patient self-reported questionnaire containing questions on age (years), sex (male/female/other), body mass (kg), and length (meters) to calculate the body mass index (BMI), current medication use non-steroidal anti-inflammatory drugs (NSAIDs), any disease-modifying anti-rheumatic drug (DMARD) (categorized into conventional DMARD, biologic DMARD, targeted synthetic DMARD), or no anti-rheumatic medication or anti-inflammatory medication used), self-reported symptom duration (years), number of joint replacements, education level (low: primary school or pre-vocational secondary education; medium: senior general secondary education or pre-university education or secondary vocational education; high: Bachelor or Master at University (of Applied Sciences)) and, if 66 years or younger, having a paid job (yes/no). Comorbidities were recorded based on a questionnaire developed by Statistics Netherlands, asking for the presence of 19 different comorbidities (yes/no) [23]. Moreover, we requested the treating rheumatologist to provide measures of disease activity in terms of the Disease Activity Score 28 (DAS-28) for RA and the Bath Ankylosing Spondylitis Disease Activity Index (BASDAI) for axSpA. These measures were collected as close as possible to the date of the participant’s enrollment in the study. All baseline data were tested for normality using the Kolmogorov–Smirnov or similar test, where appropriate.

#### Physical functioning measures

Physical functioning was measured using three different questionnaires: the Patient-Reported Outcomes Measurement Information System—Physical Function item bank 10 (PROMIS PF-10) [24] was used in both populations, the Health Assessment Questionnaire—Disability Index (HAQ-DI) [25] in people with RA and the Bath Ankylosing Spondylitis Functional Index (BASFI) in people with axSpA [26].

The PROMIS PF-10 [24] comprises ten questions from the PROMIS physical function item bank, which all are scored on a five-point scale ranging from 1 to 5 with higher scores indicating better physical functioning. The total score was calculated by uploading the data into a scoring system program [27], after which the T scores are calculated. The PROMIS PF-10 can range from 13.5 to 61.9 [28], where a higher score indicates better physical functioning. A validated Dutch version was used and, calculations of T scores were standardized to the Dutch population [29].

The HAQ-DI [25] contains 20 items concerning the ability to perform daily activities, divided over eight domains. There are four possible responses and corresponding scores for each question (without any difficulty; score = 0, with some difficulty; score 1, with much difficulty score = 2, and unable to do score = 3). The highest score reported by the patient for any component question in each domain determines the score for that domain. A validated Dutch translation of the HAQ-DI was used [30]. The total HAQ-DI score was calculated by the sum of the scores of the eight domains divided by eight, after correcting for the use of aids or devices [25]. While there is no data evidence as to what constitutes mild, moderate, or severe disability, a score of ≤ 1.0 is regarded as indicating mild disability, and a score ≥ 2.0 is considered to indicate severe disability [31].

The BASFI is a validated instrument to assess the degree of functional limitation in patients with Ankylosing Spondylitis [26]. It consists of ten questions related to activities of daily living (eight on physical functioning and two on coping with everyday life), which are all scored on a 11-point scale ranging from 0 (easy) to 10 (impossible to perform) with higher scores indicating worse physical functioning. The mean of the individual scores is calculated to give the overall BASFI score ranging from 0 (no impairment) to 10 (severe impairment), with higher scores indicating more functional limitations [26]. A Dutch translation of the BASFI was used.

#### Patient specific complaints instrument (PSC)

The PSC is a validated instrument in people with chronic diseases to identify and quantify limitations in activity [16–18]. It was administered face-to-face by a trained researcher (MvW, MT). Patients were asked to describe three activities in daily life that were currently difficult to perform and found important to improve. Thereafter, the three PSC activities were prioritized by the patients from most important to least important. Subsequently, the patient was asked to score each of the activities on an 11-point numeric rating scale (NRS) (Anchor 0: able to perform activity without any problems; 10: unable to perform activity). As half of the participants would be randomized to a control condition, participants were not asked to formulate the limited activity in terms of a treatment goal, but only in terms of limited activities they desired to improve.

#### ICF linking method

**Fig. 1:**
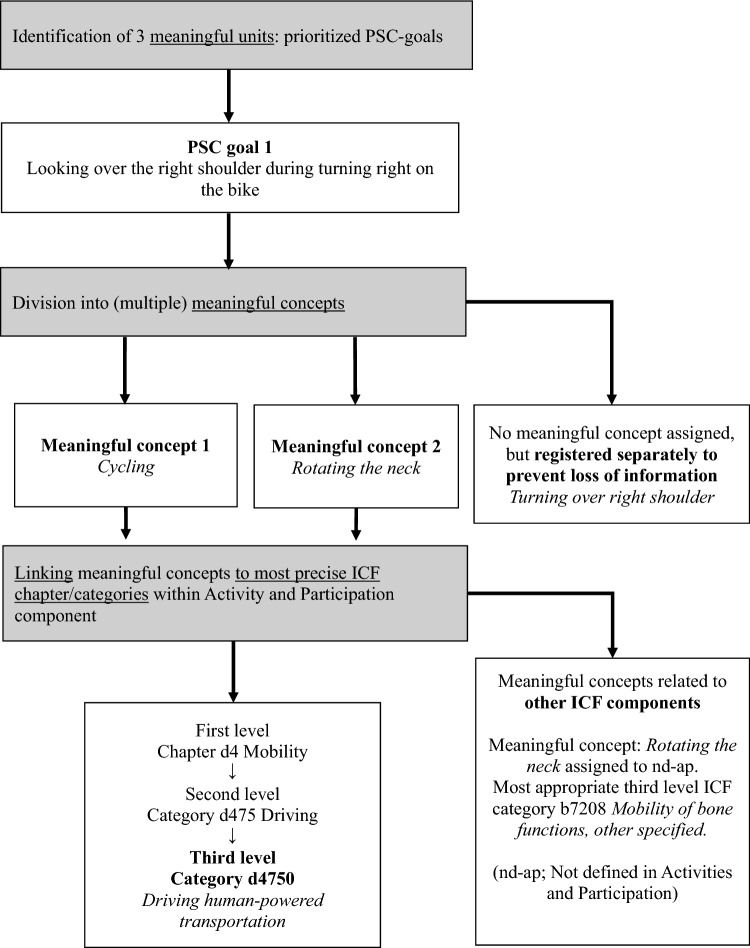
Standardized linking process of the patient specific complaints (PSC) activities to specific International Classification of Functioning, Disability and Health (ICF) categories: an example

The PSC activities were linked to the ICF following standardized linking rules [32, 33]. The linking process is shown in Fig. 1. Prior to the linking process, the researchers individually acquired knowledge of the conceptual fundamental elements of the ICF, components, chapters, categories of the detailed level classification, and definitions. Since the PSC pertains to daily activities, the linking was only done for the ICF component “Activities and Participation”. The Dutch translation of the ICF as published on the WHO website was used for the linking process (https://www.whofic.nl/familie-van-internationale-classificaties/referentie-classificaties/icf accessed 1 November 2022).

In addition to the standardized linking rules proposed by Cieza et al. [32, 33], five practical agreements were formulated to facilitate unambiguous definition of concepts and linking to ICF, which are shown in the Supplemental material.

Two researchers (MT and TD) independently performed all steps of the linking process. In case of disagreements between the two researchers, a third researcher (SvW) was consulted. In the first step, each PSC activity was divided into (multiple) relevant meaningful concepts. For example, the PSC activity: “Walking about 3000 m to the supermarket to shop groceries” was divided into two meaningful concepts: “Walking long distances” and* “*Shopping”*.* Parts of the PSC activity that could not be assigned to a meaningful concept were registered separately to prevent a loss of information. Subsequently, all identified meaningful concepts were linked to the most specific ICF category within the “Activities and Participation” component, with the first level and, where applicable, the second-level category and the third-level category representing increasingly more specific information. For example, the meaningful concept “Cycling” was linked within the first-level category (chapter) “Mobility” and the second-level category “Driving” and to the third-level category “Driving human-powered transportation”.

For the determination of the overlap with ICF Core sets, a comparison with the categories in the component “Activities and Participation” of the ICF Core Set for RA [12] and ICF Core Set for Ankylosing Spondylitis (AS) [34] was made. The ICF categories in the ICF RA and AS Core Sets are all defined at the second level. To enable a comparison between the content of the identified PSC activities and the content of the Core Sets, for the activities with a third-level ICF category, the corresponding second-level categories were used. If no ICF category was appropriate within the “Activities and Participation” component but rather another component of the ICF, this meaningful concept was assigned as “not defined in Activities and participation (nd-ap)”. For example, if a PSC activity was “Looking over the shoulder when changing direction while riding a bicycle”, the meaningful concept “Rotating the neck” was linked to the category nd-ap since the most appropriate ICF category was “Mobility of bone functions, specified”.

#### Analyses

For this analysis, we utilized baseline data from the included patients available on February 14, 2022. As of that date, the inclusion of participants in the studies was still ongoing. The target enrollment for both RCTs was set at 215 participants. Given the descriptive design of this study, in which we wanted to describe the nature of functional limitations no supplementary power calculations were performed. A minimum number of 150 patients per diagnosis group was considered sufficient to estimate both low and high frequencies of specific limitations with sufficient precision. We included all available data at the moment of the analysis as we considered it unethical to leave individuals out [35]. Descriptive analyses of the baseline characteristics were done for people with RA or axSpA separately.

For both populations, the total numbers of meaningful concepts and the numbers and frequencies of unique ICF categories were calculated in total and for each of the three ranked PSC activities separately. In addition, the mean number of ICF categories per PSC activity per participant were calculated. Finally, the overlap with the Comprehensive and Brief ICF Core Sets for RA and AS was determined by comparing the Core Set items to the uniquely identified second-level ICF categories derived from the PSC activities. All statistical analyses were performed using the Statistical Package for the Social Sciences (SPSS), Released 2017, IBM SPSS Statistics for Windows, Version 25.0. Armonk, NY, United States of America: IBM Corp.

## Results

### Demographics and disease characteristics

Table 1 shows the baseline characteristics of the 206 and 155 participants with RA or axSpA with all of the data being normally distributed. Their mean ages (SD) were 58.7 (12.9) and 53.2 (11.8) years), the proportion of females was 90.8% and 47.1% and the self-reported symptom duration was 21.6 (13.5) and 24.7 (14.4) years) in the RA and axSpA groups, respectively. More than 70% of both RA and axSpA groups had three or more comorbidities.

**Table 1:** Baseline characteristics of people with RA or axSpA and severe functional disability participating in a randomized controlled trial on longstanding exercise therapy

	RA (*N* = 206)	axSpA (*N* = 155)
Age, mean (SD)	58.7 (12.9)	53.2 (11.8)
Sex, female, *N* (%)	187 (90.8)	73 (47.1)
BMI, mean (SD)	27.6 (6.1)	28.8 (6.6)
Current medication use, *N* (%)		
Any DMARD	139 (67.5)	101 (65.2)
csDMARD	83 (59.7)	20 (19.8)
bDMARD	102 (73.4)	93 (92.1)
tsDMARDs	12 (8.6)	0 (0)
NSAIDs	90 (43.7)	79 (51.0)
No anti-rheumatic or anti-inflammatory medication	9 (4.4)	
Self-reported symptom duration (years), mean (SD)	21.6 (13.5)	24.7 (14.4)
Number of comorbidities, *N* (%)	*N* = 204	*N* = 153
0	7 (3.4)	7 (4.5)
1–2	51 (24.8)	33 (19.3)
3–4	67 (32.5)	46 (29.7)
≥ 5	79 (38.3)	67 (45.2)
Joint replacement surgeries ≥ 1, *N* (%)	80 (38.8)	25 (16.1) *N* = 154
DAS-28, mean (sd)	3.1 (1.3) *N* = 151	–
BASDAI, mean (sd)	–	5.0 (2.0) *N* = 94
Education level, *N* (%)		
Low	89 (43.2)	39 (25.2)
Medium	58 (28.2)	58 (37.4)
High	59 (28.6)	58 (37.4)
Work status, *N* (%)		
≤ 66 years old	147 (71.4)	135 (87.1)
Paid job	42 (28.6)	43 (31.9)
No job, health problems	59 (40.1)	62 (45.9)
No job, other reasons	46 (31.3)	30 (22.2)
PROMIS PF-10 (13.5–61.9), mean (SD)	33.9 (5.2)	35.8 (4.5)
HAQ-DI (0–3), mean (SD)	1.7 (0.5)	–
BASFI (0–10), mean (SD)	–	6.1 (1.9) *N* = 153

*axSpA* axial spondyloarthritis; *BASDAI* bath ankylosing spondylitis disease activity index; *BASFI* Bath Ankylosing Spondylitis Functional Index; *bDMARDs* biologic disease-modifying anti-rheumatic drugs; *BMI* body mass index; *csDMARDs* conventional synthetic disease-modifying anti-rheumatic drugs; DAS-28, Disease Activity Score 28; Education level ( Low, primary school or pre-vocational secondary education; Medium, senior general secondary education or pre-university education or secondary vocational education; High, Bachelor or Master at University (of Applied Sciences)); *HAQ-DI* Health Assessment Questionnaire Disability Index; *NSAIDs* nonsteroidal anti-inflammatory drugs; *PROMIS PF-10* patient-reported outcomes measurement information system physical function 10; *RA* rheumatoid arthritis; *tsDMARDs* targeted synthetic disease-modifying anti-rheumatic drugs

### Number of identified meaningful concepts derived from PSC, and total and unique ICF categories

Results are shown in Table 2. In total 911 and 769 meaningful concepts were identified from the PSC activities for people with RA and axSpA, respectively. These were linked to 909 and 759 ICF categories, of which 72 and 57 were unique in RA and axSpA, respectively. All uniquely identified ICF categories were on the second-level (*n* = 5 in RA and *n* = 4 in axSpA) or third-level (*n* = 67 in RA and *n* = 53 in axSpA). When all meaningful concepts were only linked to second-level categories, there were 25 and 23 unique ICF categories for RA and axSpA, respectively. There were two meaningful concepts in RA and ten in axSpA that could not be linked to an ICF category within the component “Activities and Participation” but within the component “Body functions” and were thus assigned to the “nc-ap” category.

**Table 2:** Results of PSC activities, meaningful concepts and ICF categories in people with RA or axSpA and severe functional disability

	RA (*N* = 206)	axSpA (*N* = 155)
Total PSC activities, *N*	618	465
PSC scores (0–10), mean (SD)		
PSC activity 1	7.5 (1.4)	7.8 (1.0)
PSC activity 2	7.5 (1.3)	7.6 (1.1)
PSC activity 3	7.6 (1.3)	7.4 (1.1)
Total meaningful concepts	911	769
Total meaningful concepts “nd-ap”	2	10
Total number of ICF categories	909	759
Total unique ICF categories, second level	5	4
Total unique ICF categories, third level	67	53
ICF categories per participant, mean (SD)	4.4 (0.6)	4.9 (0.8)
ICF categories per participant: 3	42	16
ICF categories per participant: 4	80	44
ICF categories per participant: 5	51	49
ICF categories per participant: 6	24	34
ICF categories per participant: 7	8	9
ICF categories per participant: 8	1	2
ICF categories per participant: 9	–	1

*axSpA* axial spondyloarthritis; *ICF* International Classification of Functioning, Disability and Health; *IQR* interquartile range; *PSC* patient-specific complaints instrument; *RA* rheumatoid arthritis

### Type and frequency of ICF categories

The total numbers of identified ICF categories in the component “Activities and Participation” and their frequencies are shown in Table 3. Regarding the distribution of the linked activities across the relevant ICF chapters, the majority of the total number of ICF categories related to the ICF chapter “Mobility”, in both RA (76.6%) and axSpA (70.1%). None of the activities appeared to be related to the ICF chapters “Learning and applying knowledge”, “General tasks or demands” or “Interpersonal interactions and relationships”.

**Table 3:** ICF categories in the component “Activities and participation” derived from PSC activities in people with RA or axSpA and severe functional disability

Code and description of ICF category	RA (*N* = 206)	axSpA (*N* = 155)
**d1 Learning and applying knowledge, *N*** **(%)**	0 (0%)	0 (0%)
➢ *d170 Writing*	0	0
**d2 General tasks and demands, *N* (%)**	0 (0%)	0 (0%)
➢ *d230 Carrying out daily routine*	0	0
➢ *d240 Handling stress and other psychological demands*	0	0
**d3 Communication, *N* (%)**	6 (0.7%)	4 (0.5%)
➢ *d345 Writing messages*	3	1
➢ *d360 Using communication devices and techniques*	3	3
• d3601 Using writing machines	3	3
**d4 Mobility,** ***N* (%)**	696 (76.6%)	532 (70.1%)
➢ *d410 Changing basic body position*	102	91
• d4100 Lying down	2	6
• d4101 Squatting	3	6
• d4102 Kneeling	0	2
• d4103 Sitting	60	30
• d4104 Standing	3	2
• d4105 Bending	22	44
• d4107 Rolling over	4	0
• d4108 Other specified	8	1
➢ *d415 Maintaining a body position*	35	89
• d4150 Maintaining a lying position	1	2
• d4151 Maintaining a squatting position	1	0
• d4152 Maintaining a kneeling position	0	1
• d4153 Maintaining a sitting position	10	26
• d4154 Maintaining a standing position	23	60
➢ *d430 Lifting and carrying objects*	47	51
• d4300 Lifting	34	42
• d4301 Carrying in the hands	7	9
• d4302 Carrying in the arms	6	0
➢ *d435 Moving objects with lower extremities*	1	0
• d4351 Kicking	1	0
➢ d440 Fine hand use	98	62
• d440 Fine hand use	0	0
• d4400 Picking up	2	4
• d4401 Grasping	51	39
• d4402 Manipulating	44	11
• d4408 Other specified	1	8
➢ *d445 Hand and arm use*	23	10
• d4452 Reaching	2	6
• d4453 Turning or twisting the hands or arms	11	4
• d4454 Throwing	2	0
• d4455 Catching	1	0
• d4458 Other specified	1	0
• d4459 Unspecified	6	0
➢ *d449 Carrying, moving, and handling objects, other specified and unspecified*^*a*^	0	0
➢ *d450 Walking*	**268**	**158**
• d450 Walking	0	0
• d4500 Walking short distances	48	12
• d4501 Walking long distances	121	92
• d4502 Walking on different surfaces	79	43
• d4508 Other specified	20	11
➢ *d451 Stair climbing*	62	32
➢ *d455 Moving around*	3	2
• d4451 Climbing	0	1
• d4552 Running	1	0
• d4554 Swimming	2	1
➢ *d460 Moving around in different locations*	7	2
• d4600 Within the home	2	2
• d4602 Outside the home and other buildings	1	0
• d4608 Other specified	4	0
➢ *d465 Moving around using equipment*	0	0
➢ *d470 Using transportation*	0	0
➢ *d475 Driving*	50	35
• d4750 Driving human-powered transportation	37	25
• d4751 Driving motorized vehicles	3	4
• d4752 Driving animal-powered vehicles	1	0
• d4758 Other specified	9	6
**d5 Self-care, *N* (%)**	69 (7.6%)	60 (7.9%)
➢ *d510 Washing oneself*	22	9
• d5100 Washing body parts	5	3
• d5101 Washing whole body	9	4
• d5102 Drying oneself	8	2
➢ *d520 Caring for body parts*	2	2
• d5202 Caring for hair	1	2
• d5204 Caring for toenails	1	0
➢ *d530 Toileting*^a^	7	7
• d5301 Regulating defecation	4	4
➢ *d540 Dressing*	35	42
• d5400 Putting on clothes	22	21
• d5401 Taking off clothes	7	2
• d5402 Putting on footwear	*6*	19
➢ *d550 Eating*	3	0
➢ *d560 Drinking*	0	0
➢ *d570 Looking after one’s health*	0	0
**d6 Domestic life, *N*** **(%)**	100 (11.0%)	136 (17.9%)
➢ *d620 Acquisition of goods and services*	19	35
• d6200 Shopping	19	35
➢ *d630 Preparing meals*	23	32
• d6300 Preparing simple meals	4	2
• d6309 Preparing meals, unspecified	19	30
• d6403 Using household appliances	29	28
➢ *d640 Doing housework*	38	44
• d6400 Washing and drying clothes and garments	1	2
• d6401 Cleaning cooking area and utensils	0	3
• d6402 Cleaning living area	6	7
• d6403 Using household appliances	29	28
• d6408 Other specified	1	4
• d6409 Unspecified	1	0
➢ *d650 Caring for household objects*	19	23
• d6501 Maintaining dwelling and furnishings	0	1
• d6503 Maintaining vehicles	2	0
• d6505 Taking care of plants, indoors and outdoors	11	17
• d6506 Taking care of animals	6	5
➢ *d660 Assisting others*	1	2
• d6609 Assisting others, unspecified	1	2
**d7 Interpersonal interactions and relationships, *N*** **(%)**	0 (0%)	0 (0%)
➢ *d760 Family relationships*	0	0
➢ *d770 Intimate relationships*	0	0
**d8 Major life areas, *N*** **(%)**	3 (0.3%)	4 (0.5%)
➢ *d845 Acquiring, keeping, and terminating a job*	0	0
➢ *d850 Remunerative employment*	0	0
➢ *d859 Work and employment, other specified and unspecified*	3	4
➢ *d859 Work and employment, other specified and unspecified*	3	4
➢ *d870 Economic self-sufficiency*	0	0
**d9 Community, social and civic life, *N*** **(%)**	36 (4.0%)	23 (3.0%)
➢ *d910 Community life*	0	0
➢ *d920 Recreation and leisure*	36	23
• d9200 Play	1	0
• d9201 Sports	8	3
• d9202 Arts and culture	8	0
• d9203 Crafts	1	0
• d9205 Socializing	5	5
• d9208 Other specified	3	0
• d9209 Unspecified	10	15

*axSpA* axial spondyloarthritis; *ICF* International Classification of Functioning, Disability and Health; *PSC* patient specific complaints instrument; *RA* rheumatoid arthritis

^a^ The ICF category for d530 toiling comprised a total 7 for RA and for axSpA. Four times for both RA and axSpA the ICF category d4531 was assigned and 3 times for both RA and axSpA d530 was assigned

Table 4 summarizes the five most frequently identified ICF categories based on the meaningful concepts of all three PSC activities combined and per PSC activity separately. For all PSC activities combined, the five most frequent activities related to “Walking” (RA and axSpA both 2: “Walking long distances” and “Walking on different surfaces”), “Changing basic body position (sitting (RA) and bending” (axSpA)), “Stair climbing” (RA), “Grasping” (RA), “Maintaining a standing position” (axSpA), and “Lifting” (axSpA).

**Table 4:** Five most prevalent ICF categories identified in people with RA or axSpA and severe functional disability, in total and by PSC activity

RA (*N* = 206)			
Ranking	ICF code	Total three PSC activities (*N* = 909 ICF categories)	Number of ICF categories (%)
1	d4501	Walking long distances	121 (13.3)
2	d4502	Walking on different surfaces	79 (8.7)
3	d451	Stair climbing	62 (6.8)
4	d4103	Changing basic body position: sitting	60 (6.6)
5	d4401	Grasping	51 (5.6)

Ranking	ICF code	PSC activity 1 (*N* = 316 ICF categories)	Number of ICF categories (%)
1	d4501	Walking long distances	75 (23.7)
2	d4502	Walking on different surfaces	47 (14.9)
3	d4500	Walking short distances	33 (10.4)
4	d4401	Grasping	14 (4.4)
5	d4103	Changing basic body position: sitting	12 (3.8)

Ranking	ICF code	PSC activity 2 (*N* = 294 ICF categories)	Number of ICF categories (%)
1	d4501	Walking long distances	28 (9.5)
2	d451	Stair climbing	25 (8.5)
3	d4103	Changing basic body position: sitting	20 (6.8)
4	d4750	Driving human-powered transportation	19 (6.4)
5	d4401	Grasping	18 (6.1)

Ranking	ICF code	PSC activity 3 (*N* = 299 ICF categories)	Number of ICF categories (%)
1	d4103	Changing basic body position: sitting	28 (9.4)
2	d451	Stair climbing	27 (9.0)
3	d4402	Manipulating	20 (6.7)
4	d4401	Grasping	19 (6.4)
5	d4501	Walking long distances	18 (6.0)

AxSpA (*N* = 155)			
Ranking	ICF code	Total three PSC activities (*N* = 759 ICF categories)	Number of ICF categories (%)
1	d4501	Walking long distances	92 (12.1)
2	d4154	Maintaining a standing position	60 (7.9)
3	d4105	Changing basic body position: bending	44 (5.8)
4	d4502	Walking on different surfaces	43 (5.7)
5	d4300	Lifting	42 (5.5)

Ranking	ICF code	PSC activities 1 (*N* = 247 ICF categories)	Number of ICF categories (%)
1	d4501	Walking long distances	50 (20.2)
2	d4502	Walking on different surfaces	22 (8.9)
3^a^	d4154 /	Maintaining a standing position /	17 (6.9)
	d451	Stair climbing	17 (6.9)
5^a^	d4300 /	Lifting /	10 (4.0)
	d4153	Maintaining a sitting position	10 (4.0)

Ranking	ICF code	PSC activities 2 (*N* = 253 ICF categories)	Number of ICF categories (%)
1	d4154	Maintaining a standing position	30 (11.9)
2^a^	d4105 /	Changing basic body position: bending	19 (7.5)
	d4501	Walking long distances	19 (7.5)
4	d4401	Grasping	16 (6.3)
5	d4750	Driving human-powered transportation	14 (5.5)

Ranking	ICF code	PSC activities 3 (*N* = 259 ICF categories)	Number of ICF categories (%)
1	d4501	Walking long distances	23 (8.9)
2	d4300	Lifting	20 (7.7)
3^a^	d6200 /	Shopping	16 (6.2)
	d4105	Changing basic body position: bending	16 (6.2)
5	d4103	Changing basic body position: sitting	15 (5.8)

*axSpA* axial spondyloarthritis; *ICF* International Classification of Functioning, Disability and Health; *PSC* patient specific complaints instrument; *RA* rheumatoid arthritis

^a^Shared ranking in top-5 due to same reported number

The five most common ICF categories identified based on the separate PSC activities, showed a high agreement, but additionally identified “Driving human-powered transportation” (RA and axSpA), “Manipulating” (RA), “Walking short distances” (RA), “Shopping” (axSpA), “Grasping” (axSpA), “Maintaining a sitting position” (axSpA), and “Changing basic body position: sitting” (axSpA).

When comparing the frequencies of ICF categories across the three ranked activities, limitations in “Walking” were relatively more frequent in the PSC activities ranked 1, in both RA and axSpA. In RA “Changes in basic body position: sitting”, “Grasping”, and ‘Manipulating” were relatively more frequent in activities ranked 2 or 3, whereas in axSpA “Changing basic body position: sitting”, “Changing basic body position: bending” and “Lifting” were relatively more frequent in activities ranked 2 or 3.

### Overlap and differences between identified ICF categories and the Brief and Comprehensive ICF Core Sets

An overview of the overlap and differences between the identified ICF categories and the Comprehensive and Brief ICF Core Sets for RA and axSpA within the “Activities and Participation” component is presented in Table 5. The Comprehensive Core Set for RA consists of 32 second-level categories of which 21 (66%) were present in this study. The Brief Core Set for RA consists of six items of which four (67%) were present in this study. Of the 25 identified second-level ICF categories in our study, four categories were not included in the Core Sets for RA: “Stair climbing”, “Writing messages”, “Moving objects with lower extremities”, and “Caring for household objects” with “Stair climbing” being the most common (62/909 total number of ICF categories, 6.8%).

**Table 5:** ICF Core Sets categories in the component “Activities and Participation” and their overlap with the ICF categories derived from PSC activities in people with RA or axSpA and severe functional disability

ICF category	RA (*N* = 206)	ICF Core Sets RA	axSpA (*N* = 155)	ICF Core Sets AS
d170 Writing	–	*	–	
d230 Carrying out daily routine	–	**	–	**
d240 Handling stress and other psychological demands	–		–	*
d345 Writing messages	+		+	
d360 Using communication devices and techniques	+	*	+	
d410 Changing basic body position	+	**	+	**
d415 Maintaining a body position	+	*	+	*
d430 Lifting and carrying objects	+	*	+	*
d435 Moving objects with lower extremities	+		–	
d440 Fine hand use	+	**	+	
d445 Hand and arm use	+	**	+	
d449 Carrying, moving, and handling objects, other specified and unspecified	–	*	–	
d450 Walking	+	**	+	**
d451 Stair climbing	+		+	
d455 Moving around	+	*	+	*
d460 Moving around in different locations	+	*	+	
d465 Moving around using equipment	–	*	–	
d470 Using transportation	–	*	–	*
d475 Driving	+	*	+	**
d510 Washing oneself	+	*	+	*
d520 Caring for body parts	+	*	+	*
d530 Toileting	+	*	+	*
d540 Dressing	+	*	+	*
d550 Eating	+	*	–	
d560 Drinking	–	*	–	
d570 Looking after one’s health	–	*	–	*
d620 Acquisition of goods and services	+	*	+	*
d630 Preparing meals	+	*	+	
d640 Doing housework	+	*	+	*
d650 Caring for household objects	+		+	
d660 Assisting others	+	*	+	*
d760 Family relationships	–	*	–	**
d770 Intimate relationships	–	*	–	*
d845 Acquiring, keeping, and terminating a job	–		–	**
d850 Remunerative employment	–	**	–	**
d859 Work and employment, other specified and unspecified	+	*	+	
d870 Economic self-sufficiency	–		–	*
d910 Community life	–	*	–	*
d920 Recreation and leisure	+	*	+	**
ICF Categories in Comprehensive ICF Core Set		32		24
Total number of second-level ICF categories	25		23	
Overlapping ICF categories with Comprehensive Core Set	21		14	
ICF categories in Brief ICF Core Set		6		8
Overlapping ICF categories with Brief Core Set	4		4	

*AS* ankylosing spondylitis; *axSpA* axial spondyloarthritis; *ICF* International Classification of Functioning, Disability and Health; *RA* rheumatoid arthritis

− Not included in our study population

+ Included in our study population

*Included in the Comprehensive Core Set

**Included in the Brief and Comprehensive Core Set

The Comprehensive Core Set for AS comprises of 24 second-level categories of which 14 were reported in this study (58%). The Brief Core Set for AS consists of eight items of which four (50%) were present in this study. Of the 23 identified second-level categories, nine categories were not included in the Core Sets for AS: “Fine hand use”, “Writing messages”, “Using communication devices and techniques”, “Hand and arm use”, “Moving around in different locations”, “Preparing meals”, “Caring for household objects”, “Work and employment, other specified and unspecified”, and “Stair climbing” with “Fine hand use” being most frequent (62/759 total number of ICF categories, 8.2%).

## Discussion

The most frequent limitations in activities as prioritized by people with RA or axSpA and severe functional disability concerned the ICF chapter “Mobility”, in particular the categories related to “Walking” and “Changing basic body position”. In RA, other frequent limitations were related to “Grasping” and “Stair climbing” whereas in axSpA this concerned “Lifting” and “Maintaining a standing position”. There was considerable overlap between the ICF categories identified in the study populations and the corresponding ICF Core Sets, to a greater extent in RA than in axSpA. In our study population, thirteen ICF categories (four in RA and nine in axSpA) were identified that were not included in the Comprehensive Core Sets for RA/AS. Among these categories, “Stair climbing” for RA and “Fine hand use” for axSpA demonstrated a prevalence of more than 5%.

Our findings are partly in line with a previous study employed in four different countries linking rehabilitation goals to the ICF in people with RA patients, where within the “Activities and Participation” component “Walking” and “Self-care” reported most [10]. Activities such as “Stair climbing” and “Changing basic body position” were frequently reported in our population but were not found in the latter study. The previous study did not include patients with axSpA, whereas the inclusion of two populations within our study enabled the comparison among people with different rheumatological diagnoses.

Other comparisons are difficult to make, as the population, setting and methods in the present study importantly differed from the previous study [10]. In the present study, the included participants had more functional disability as shown by higher HAQ-DI scores, and were treated in primary care and not in a multidisciplinary rehabilitation setting. Moreover, a different method for the assessment of treatment goals was used with elicitation and prioritization of limited activities without explicit goal-setting and only pertained to one component of the ICF (i.e. “Activities and Participation”). Our study also included people with axSpA, in which knowledge on this topic is more limited. A study in veterans with spondyloarthritis (SpA, including AS) explored the relation between the disease and physical function [36] by means of a survey. They found veterans with SpA had significant more limitations in “Walking”, “Transferring”, and “Dressing” [36]. Although, this study did not use treatment goals, the findings are similar to our study.

The content of the Brief and Comprehensive Core Sets for RA or AS were well reflected in the prioritized activities. Overall, more than half of linked ICF categories as derived from the prioritized limited activities corresponded with the contents of the ICF Core Sets for RA or AS. However, there were exceptions in our study, where certain categories such as “Carrying out daily routine”, “Remunerative employment”, “Family relationships”, and “Acquiring, keeping, and terminating a job” were included in the Brief and Comprehensive Core Sets for AS but were not identified in our study populations. Similarly, for RA, the ICF categories “Carrying out daily routine” and “Remunerative employment” were part of the Brief and Comprehensive Core Sets for RA but were not identified in our study populations. A possible reason for the absence of these categories in our populations could be due to participants being requested to identify three specific limitations in activities that were found important and could be improved with an intervention such as exercise therapy. The discrepancies between the nature of limited activities seen in the present study and the content of the corresponding ICF Core Sets may warrant further exploration. It is first of all possibly related to the specific selection of the study population, being a population of people with severe functional disability. Moreover, the ICF Core Sets were developed more than 10 years ago (RA in 2004 and AS in 2010). Due to the developments of pharmacological interventions in recent years and changing needs of society, limitations in activity might also have evolved over time. Furthermore, for people with axSpA, only the ICF Core Sets for AS were available, whereas the axSpA population encompasses both radiographic and non-radiographic axSpA with patients possibly facing other challenges in daily activities.

This study has a number limitations. First, as our study concerned baseline data of RCTs with specific inclusion and exclusion criteria, it thus concerns a selected population. Moreover, as our RCTs pertained to long standing exercise therapy, patients with a relatively positive attitude towards exercise therapy may have been overrepresented. Either or not related to the previous points, the proportion of females was relatively high in our population, whereas it is known that women are in general more willing to participate in research than men [37]. Second, this study concerned the ICF component “Activities and Participation” only and we can, therefore, not make assumptions on limitations perceived regarding the other ICF components. Finally, despite the elaborate descriptions of the methods for linking goals to the ICF as proposed by Cieza et al. [32, 33], it was in some cases challenging to link free text of PSC activities to the most appropriate ICF category. For example, PSC activities did not always contain enough specific information to determine the most precise category resulting in the ICF category unspecified. Accurately setting treatment goals can be challenging, because it refers to a future state of functioning achieved through planned treatment actions. A PSC activity that does not contain enough information to determine the most precise ICF category highlights the need for more training of healthcare professionals on goal-setting to further improve the use of PSC activities for individualized tailored treatment of people with RA or axSpA. To overcome some of these problems, some adaptions or additions to the existing linking rules may facilitate unambiguous definition of meaningful concepts and linking to the ICF.

In conclusion, to our knowledge, this is the first study describing the nature of functional limitations as assessed with the PSC for people with RA or axSpA and severe functional disability. It provides insight into the nature and most frequent functional limitations in this subgroup within the “Activities and Participation” component of the ICF, and can, therefore, facilitate healthcare professionals in identifying individual functional limitations in activities and participation and thus improving treatment. The overlap with the Core Sets for RA and AS was relatively high, however, clinicians should be aware that not all RA or AS Core Sets items are prevalent in practice and some prevalent activity limitations prioritized by individual patients are not included in the ICF Core Sets.

### Supplementary Information

Below is the link to the electronic supplementary material.

Supplementary file1 (DOCX 30 KB)

## Data Availability

The data underlying this article will be shared on reasonable request to the corresponding author.
